# Cardiac Rehabilitation in 2026: Bridging Guideline Mandates, Patient Realities and Emerging Innovations

**DOI:** 10.3390/jcm15041362

**Published:** 2026-02-09

**Authors:** Tomasz Rechciński, Barbara Bralewska, Aleksandra Rechcińska

**Affiliations:** 1 1st Department of Cardiology, Medical University of Lodz, 91-347 Lodz, Poland; 2 SP ZOZ Central Clinical Hospital, Medical University of Lodz, 92-213 Lodz, Poland; 3 Independent Researcher, 90-014 Lodz, Poland

We are pleased to introduce the Special Issue of the Journal of Clinical Medicine, dedicated to “Recent Clinical Advances in Cardiac Rehabilitation”, which addresses one of the most critical topics in secondary prevention of cardiovascular diseases.

In the Brothers Grimm’s timeless fairy tale, a prince trapped in the body of a frog awaits a single transformative kiss to reclaim his true form. Cardiac rehabilitation has long resembled this enchanted prince: a therapy with royal evidence behind it, yet still confined to a marginal, often overlooked place in routine care. What it now awaits is not magic, but the deliberate “kiss” of full implementation—across regions, cultures and health systems—that would finally allow its true potential to emerge.

Contemporary guidelines from both Europe and Asia leave little doubt about the central role of cardiac rehabilitation in secondary prevention. The 2021 ESC Guidelines on cardiovascular disease prevention in clinical practice explicitly position structured rehabilitation as a core component of integrated risk-factor management, lifestyle modification and long-term follow-up after major cardiovascular events [1]. Similarly, consensus recommendations from Japanese expert groups emphasise that every patient after myocardial infarction should be offered a comprehensive, multidisciplinary rehabilitation programme, combining exercise training, education, psychological support and vocational counselling as a standard of care rather than a luxury [2]. Together, these documents set a clear, global benchmark: high-quality cardiac rehabilitation is no longer optional—it is a clinical imperative.

Yet, as so often in cardiovascular prevention, what is written in guidelines is not what patients receive in everyday practice. Recent data from the INTERASPIRE programme paint a sobering picture of secondary prevention and cardiac rehabilitation worldwide, showing that a large proportion of patients with established coronary heart disease are neither referred to, nor enrolled in, structured rehabilitation, and that achievement of lifestyle and risk-factor targets remains disappointingly low. Despite robust evidence and strong recommendations, many patients leave hospital without a clear path towards sustained physical activity, smoking cessation, weight control or psychosocial support [3].

Reports from China echo this paradox in a rapidly transforming healthcare system. Although the epidemiological burden of cardiovascular disease is immense and awareness of secondary prevention is growing, participation in supervised exercise-based rehabilitation and long-term adherence to lifestyle changes remain limited, mirroring the global pattern of underuse and unmet need [4]. In both international and national registries, the gap between the “ideal world” of guidelines and the “real world” of practice is striking—and it is this gap that continues to keep cardiac rehabilitation in its frog-like state, far from the royal status it deserves.

The COVID-19 pandemic unexpectedly acted as a stress test—and, paradoxically, as an accelerator—for cardiac rehabilitation. When centre-based programmes were abruptly interrupted or severely restricted, clinicians and patients were forced to experiment with new ways of delivering exercise training, education and psychosocial support at a distance. This disruption revealed how fragile our reliance on in-person models had been, but it also highlighted the adaptability of rehabilitation when digital tools, telemonitoring and home-based protocols are embraced [5].

In this context, several contributions in the present Special Issue offer concrete examples of how innovation can preserve—and sometimes even enhance—access to rehabilitation despite structural barriers. One study explores a structured telerehabilitation pathway that combines remote supervision, education and regular feedback to maintain exercise intensity and adherence in high-risk patients who cannot attend traditional centre-based programmes (Contributor 1). Another article examines hybrid models that blend on-site assessments with technology-assisted home sessions, showing that such approaches can sustain functional gains and risk-factor control while reducing travel burden and improving convenience (Contributor 2). Together, these works suggest that the pandemic may have been the long-awaited external shock that pushed cardiac rehabilitation closer to the transformative “kiss” of technology-enabled care.

Beyond simple telemonitoring and video visits, more technologically advanced models are beginning to emerge. A recent study on technology-assisted cardiac rehabilitation in patients with coronary heart disease, for example, integrates wearable sensors, mobile applications and structured remote coaching into a cohesive programme that delivers supervised exercise, behavioural support and risk-factor management outside the walls of the hospital. Such interventions move beyond emergency solutions and start to define a new standard of care in which digital tools are not a temporary substitute, but an integral layer of rehabilitation delivery [6].

The notion that cardiac rehabilitation can be “augmented” rather than merely “reproduced” by technology resonates strongly with the themes of this Special Issue. By systematically embedding monitoring, feedback and personalised adjustment into patients’ daily lives, higher-level technological solutions may help address long-standing problems of adherence, equity and scalability. The question is no longer whether technology can support rehabilitation, but how to design, evaluate and implement these programmes so that their benefits reach the patients who need them most

At the same time, cardiac rehabilitation must widen its gaze beyond the “typical” post-myocardial infarction or post-revascularisation patient. Emerging evidence underscores the needs of groups that have historically been underrepresented in programmes and trials—including patients with congenital heart disease, frail older adults, those with multimorbidity, and individuals from socioeconomically disadvantaged or rural backgrounds. Studies of adolescents and adults with congenital heart disease, for example, show that supervised rehabilitation is both feasible and safe in this population, improving exercise capacity and quality of life, yet referral remains the exception rather than the rule (Contributor 3).

A recent review further highlights how these “niche” populations often fall through the cracks of standard rehabilitation pathways, despite having much to gain from tailored interventions, flexible delivery models and closer integration with specialist care. Addressing their needs will require not only technical innovation, but also a shift in mindset: from a narrow, procedure-based view of eligibility towards a life-course, person-centred concept of cardiac rehabilitation as an adaptable framework for long-term cardiovascular health. Only then will the promise of rehabilitation extend to all those who could benefit from it—not just to the easiest patients to reach.

One of the most striking contributions in this issue is the report on a hybrid cardiac rehabilitation and symptom scoring programme for patients with inappropriate sinus tachycardia and postural orthostatic tachycardia syndrome referred for sinus node–sparing hybrid ablation (Contributor 4). These predominantly young women, often experiencing years of debilitating symptoms and diagnostic uncertainty, are almost invisible in traditional cardiac rehabilitation literature, yet here they are offered a structured, multidisciplinary pathway that combines early mobilisation, psychological support, respiratory therapy and home-based telerehabilitation. The high completion rates, marked reduction in Malmö POTS scores and favourable safety profile illustrate how thoughtfully designed rehabilitation can transform care even in highly specialised, previously neglected niches of cardiovascular medicine.

Another article in this issue focuses on women with heart failure, a group that is doubly disadvantaged: at higher risk of adverse outcomes, yet less likely to participate in cardiac rehabilitation (Contributor 5). The study highlights how traditional programmes often collide with the realities of women’s daily lives—caregiving duties, household responsibilities and inflexible schedules—and how symptoms of anxiety, low mood or a tendency to prioritise others over themselves further reinforce avoidance or early dropout from rehabilitation. By documenting these gendered barriers in detail and linking them to concrete participation patterns, the authors remind us that improving access for women with heart failure will require not only medical optimisation, but also redesign of rehabilitation models that acknowledges their social roles, time constraints and psychological profiles.

Psychological factors are not a decorative add-on to cardiac rehabilitation; they are often the hidden engine that determines whether patients engage with, persevere in and ultimately benefit from the programme [7]. Recent work comparing different psychological interventions within multidisciplinary rehabilitation shows that targeted approaches such as individual counselling can meaningfully reduce anxiety and depression and improve illness perception, thereby creating the emotional and cognitive space needed for patients to change behaviour and adhere to long-term risk-factor management.

In contrast, studies examining clinical, anthropometric and lifestyle determinants of enrolment remind us that many decisions about rehabilitation are also shaped by more visible variables—age, comorbidities, symptom burden, obesity, smoking, as well as social factors such as distance to the centre or competing obligations. Patients with more severe heart failure, multiple coexisting conditions or lower functional capacity may be less frequently referred or may decline participation, while those with higher education and better baseline health literacy are more likely to enrol (Contributor 6). Bringing these psychological and clinical perspectives together underscores a simple truth: improving uptake of cardiac rehabilitation requires us to address both what patients feel and believe, and the structural realities that make joining a programme either feasible or impossible.

The picture would be incomplete without considering the role of family. A dedicated review from this Special Issue synthesises evidence showing that involving partners and relatives in cardiac rehabilitation can enhance communication with clinicians, improve patients’ understanding of their condition and, in some studies, even reduce mortality and improve quality of life by strengthening emotional and practical support. At the same time, it cautions that overly intrusive involvement may threaten patients’ sense of autonomy and privacy, underscoring the need for carefully negotiated boundaries so that family members become empowering allies rather than an additional source of pressure during recovery (Contributor 7).

Beyond formal psychological counselling, softer, often underestimated practices such as meditation and yoga are beginning to find their place alongside exercise and education in cardiac rehabilitation. In a recent pilot trial, adding transcendental meditation or yoga to standard post-surgical rehabilitation did not measurably alter retinal microcirculation, yet it underscored the feasibility of integrating such approaches into routine programmes and pointed towards their potential to reduce stress, modulate autonomic tone and support patients’ overall sense of well-being (Contributor 8). When viewed together with broader data on mindfulness-based interventions in cardiovascular disease, these findings suggest that cultivating calm and attentional presence may be more than a pleasant extra—it may become a subtle but meaningful ally in helping patients sustain lifestyle change and cope with the long journey of recovery.

At the opposite end of the spectrum, another contribution in this issue descends to the cellular and subcellular consequences of exercise-based rehabilitation. By synthesising current knowledge on mitochondrial bioenergetics, this review links routine training prescriptions—frequency, intensity, interval structure—with mechanisms such as mitochondrial biogenesis, dynamics and mitophagy, arguing that at least part of the clinical benefit of rehabilitation is written into the way cardiomyocytes generate and recycle energy. In doing so, it offers a powerful reminder that when we prescribe a walking test or an interval session, we are not only improving peak oxygen uptake or six-minute walk distance, but also, quite literally, re-educating the heart at the level of its smallest power plants (Contributor 9).

Taken together, the works assembled in this Special Issue show cardiac rehabilitation in all its complexity: grounded in strong international guidelines, yet still underused; capable of adapting through digital and hybrid models; sensitive to gender, psychology and family dynamics; and reaching from social context down to mitochondrial function. Like the frog in the Brothers Grimm fairy tale, rehabilitation no longer needs to prove its royal lineage—the evidence is already there. What it now awaits is the collective “kiss” of implementation: clinicians who routinely refer, patients who are invited and supported to attend, policymakers who fund and prioritise programmes, and researchers who continue to refine and personalise interventions so that no group is left behind. When these elements finally come together, cardiac rehabilitation may at last shed its disguise and step fully into its rightful role as a central, visible, and irresistible part of modern cardiovascular care.
